# A General Deep Learning Method for Computing Molecular Parameters of a Viscoelastic Constitutive Model by Solving an Inverse Problem

**DOI:** 10.3390/polym15173592

**Published:** 2023-08-29

**Authors:** Minghui Ye, Yuan-Qi Fan, Xue-Feng Yuan

**Affiliations:** Institute for Systems Rheology, Guangzhou University, No. 230 West Outer Ring Road, Higher Education Mega-Center, Panyu District, Guangzhou 510006, China

**Keywords:** machine learning, inverse problem, deep neural network, constitutive equation, polymeric fluids, viscoelasticity

## Abstract

Prediction of molecular parameters and material functions from the macroscopic viscoelastic properties of complex fluids are of great significance for molecular and formulation design in fundamental research as well as various industrial applications. A general learning method for computing molecular parameters of a viscoelastic constitutive model by solving an inverse problem is proposed. The accuracy, convergence and robustness of a deep neural network (DNN)-based numerical solver have been validated by considering the Rolie-Poly model for modeling the linear and non-linear steady rheometric properties of entangled polymer solutions in a wide range of concentrations. The results show that as long as the DNN could be trained with a sufficiently high accuracy, the DNN-based numerical solver would rapidly converge to its solution in solving an inverse problem. The solution is robust against small white noise disturbances to the input stress data. However, if the input stress significantly deviates from the original stress, the DNN-based solver could readily converge to a different solution. Hence, the resolution of the numerical solver for inversely computing molecular parameters is demonstrated. Moreover, the molecular parameters computed by the DNN-based numerical solver not only reproduce accurately the steady viscoelastic stress of completely monodisperse linear lambda DNA solutions over a wide range of shear rates and various concentrations, but also predict a power law concentration scaling with a nearly same scaling exponent as those estimated from experimental results.

## 1. Introduction

Complex fluids, such as self-assembled surfactant and polymer solutions, exhibit dynamic instabilities and highly nonlinear flow effects owing to that their characteristic length and time scales are usually much larger than those of simple liquids. Similar to Newtonian fluids in a high Reynolds (*Re*) number flow regime, complex fluids could show turbulence-like instabilities. However, at low *Re* numbers and in the high Weissenberg (*Wi*) number flow regime, elastic forces dominate over viscous forces. This intriguing flow phenomenon observed from micro- to macro-flows has been coined as *Elastic Turbulence* [1,2,3] and has already been widely exploited in many industrial applications. For instance, it significantly improves mass transfer (mixing) efficiency in microchannels [2], greatly enhances secondary and tertiary crude oil recovery in microporous rocks [4], and nearly quadruples the heat transfer efficiency in microfluidic devices [5,6].

On the contrary, the phenomenon known as *Turbulent Drag Reduction* occurs at a high *Re* number and a moderate *Wi* number flow regime. In this scenario, the resistance of a polymer solution in pipe flows can be significantly lower than that of a Newtonian fluid with the same viscosity [7,8,9]. Thus, *Elastic Turbulence* and *Turbulent Drag Reduction* can be considered as two extreme manifestations of the non-linear dynamics of polymer solutions.

The overriding challenge in effectively regulating turbulent flow in engineering processes relies on a better understanding of strongly correlated dynamic processes with a hierarchy of characteristic length and time scales. Molecular interactions control the equilibrium microstructure, which forms on mesoscopic length scales. This, in turn, interacts with the hydrodynamic fields, which are essentially at a macroscopic scale.

The essential physics of complex fluids lie in the constitutive relationship, which forms a bridge between flow behavior and microstructure evolution during flow. The Doi–Edwards molecular theory on polymer dynamics [10] represents a milestone breakthrough in predicting the complex rheological behavior of entangled monodisperse polymer melts [11,12]. This theory makes it possible to systematically optimize the performance of polymer products from the molecular to mesoscopic levels.

In the era of digital manufacturing, it is often necessary to find solutions to inverse problems. This is carried out not only to optimize the molecular and formulation design of polymeric fluids for given rheological properties but also to identify the control variables for regulating their dynamically varying properties across multiple scales. This process even guides the discovery of new physics [13] for constructing or improving constitutive models with high physical fidelity. At least two nontrivial inverse problems exist. The first is to solve the constitutive equation inversely, which is often expressed in an implicit functional form with a high-dimensional parameter space [14]. This is carried out to estimate its control variables or material functions from the known rheometric properties. The second problem is to construct a constitutive model based on discrete datasets of polymer properties, as well as physical principles.

The present work aims to develop a general inverse learning method for solving the first problem and also aims to establish a foundation for solving the second.

Various methods have been proposed to estimate the parameters of physical models. A random search algorithm [15] used in turbulence simulation requires a large computational resource and does not guarantee good convergence. An alternative approach [16,17,18] is to directly map the outcomes of a physical model to its parameters by training a deep neural network (DNN) using a series of input parameters and model outputs. This can then be used to obtain implicit molecular parameters. Raissi et al. [19] developed physics-informed neural networks (PINN) and successfully applied them to estimate material parameters of the *Navier–Stokes* equation and the unknown pressure field from observed velocity fields. The loss functions of PINNs are constructed by imposing the conservation laws of fluid dynamics, expressed in partial differential equations (PDEs), as constraints of the machine learning process.

Reyes et al. [20] extended the PINN method to learn the effective viscosity of generalized Newtonian fluids from experimental measurements of velocity and pressure fields in time-dependent three-dimensional flows. Xu et al. [21] formulated a physics-constrained inverse learning method for modeling viscoelastic properties from observed displacement data. Mahmoudabadbozchelou et al. [22] proposed a multifidelity neural network (MFNN) architecture for data-driven, physics-informed constitutive metamodeling of complex fluids. Their results show that the MFNN algorithm outperformed purely data-driven classical DNNs and could capture the effects of experimental temperature, salt concentration, as well as aging within and outside the range of training data parameters on the rheometric properties of complex fluids.

Recently, Wang et al. [23] used a machine learning model to fit the constitutive model and subsequently performed parameter search with the trained model. Niaki et al. [24] used DNNs to predict the tensile strength of polymer concrete with experimental data. Schulte et al. [25] developed a hybrid multi-stage optimization framework to optimize material parameters using a machine learning model. More broadly, Ivanov et al. [26] proposed a method for programmable soft-matter electronics by training a multilayer perception model to determine the composition of hydrogels and automatically set the desired architecture of electronic components. Chen et al. [27] developed models combining artificial neural networks and the group contribution method to describe the binodal curve behavior of polymer-electrolyte aqueous two-phase systems and predict the partition of biomolecules.

The intrinsic properties of complex fluids and the dominant factors for controlling fluid responses to external forces are often quantified experimentally under well-defined rheometric flow conditions, either in simple shear or extensional deformation. Constitutive models can then be constructed and validated for reliable prediction of complex flows. In this paper, a DNN will be proposed and trained using a physics-informed molecular constitutive equation over its parameter space to model the steady rheometric properties of monodisperse polymer fluids.

A general method is proposed to solve an inverse problem for estimating the model parameters of the molecular constitutive model. The method is then validated by computing the model parameters from a series of steady rheometric data for entangled completely monodisperse linear lambda DNA solutions. Although the proposed DNN-based method could, in principle, be used to model the transient viscoelastic properties of entangled polymer solutions and melts, it is necessary to first demonstrate the feasibility and effectiveness of the proposed method in modeling their steady-state viscoelastic properties in this rapid communication. A more comprehensive training and validation will be left for future work.

The rest of the article is organized as follows. The problem setting is described in Section 2. The methodology is presented in Section 3. The results and discussion are reported in Section 4. The conclusions are drawn in Section 5.

## 2. Problem Setting

A general viscoelastic constitutive equation can be written in the form

(1) σ(t)=Ψ[D(t);Θ],

where σ(t) is a general viscoelastic stress tensor. It is a functional of the strain rate tensor D(t) or the strain history tensor at any instant of physical time *t*, along with a set of physical model parameters $\Theta =[\theta_{1},\theta_{2},\ldots ,\theta_{n}]$ that defines a specific complex fluid.

Instead of computing the stress response σ(t) from a set of known model parameters Θ and a specified D(t), the inverse problem involves solving for the unknown model parameters Θ of the given constitutive equation Ψ using a set of known data for σ(t) and D(t) obtained, for instance, from rheometric experiments.

As an illustration example without loss of any generality, the Rolie-Poly model [28] for modeling dynamics of entangled polymer and wormlike micellar fluids is considered here. It is simplified from the full Doi-Edwards molecular theory [10,11] and could account for the main dynamic mechanisms of entangled polymeric fluids, including reptation, convective constraint release, chain stretch etc. The conformation tensor of polymer chain A(t) under a velocity field u evolves in time as

(2) $$\frac{DA}{Dt}=L\cdot A+A\cdot L^{T}+f\left(A\right),$$

where $\frac{DA}{Dt}$ is the material derivative, L=∇u is the velocity gradient tensor, and $L^{T}$ is its transpose. For the non-Gaussian version of the Rolie-Poly model [29], which considers the finite extensibility of polymer chains, the model function f(A) is defined as

(3) $$\begin{array}{cc} f(A)= & -\frac{1}{\tau_{D}}\left(A-I\right)-\frac{2}{\tau_{R}}k_{s}\left(\lambda \right)\left(1-\sqrt{\frac{3}{\mathrm{tr}A}}\right)\left[A+\beta {\left(\frac{\mathrm{tr}A}{3}\right)}^{\delta }\left(A-I\right)\right], \end{array}$$

where I is the unit tensor, $\tau_{D}$ is the reptation time or disengagement time of the polymer chain, $\tau_{R}$ is the longest Rouse time or stretch time of the polymer chain, β is the coefficient of convective constraint release, $k_{s}\left(\lambda \right)$ is the nonlinear spring coefficient and is approximated by

(4) $$k_{s}\left(\lambda \right)=\frac{\left(3-\lambda^{2}/\chi_{max}^{2}\right)\left(1-1/\chi_{max}^{2}\right)}{\left(1-\lambda^{2}/\chi_{max}^{2}\right)\left(3-1/\chi_{max}^{2}\right)},$$

where $\lambda =\sqrt{\mathrm{tr}A/3}$ is the chain stretch ratio with respect to its equilibrium conformation, and $\chi_{max}$ is the fixed maximum stretch ratio of chain molecules. Thus, the polymeric stress can be calculated from its conformation tensor A(t) as

(5) $$\sigma_{p}\left(t\right)=Gks\left(\lambda \right)\left[A\left(t\right)-I\right]=\frac{\eta_{p}}{\tau_{D}}k_{s}\left(\lambda \right)\left[A\left(t\right)-I\right],$$

where $G=\frac{\eta_{p}}{\tau_{D}}$ is the plateau modulus and $\eta_{p}$ is the zero-shear polymer viscosity. The total stress tensor can be written as

(6) $$\sigma \left(t\right)=2\eta_{s}D\left(t\right)+\sigma_{p}\left(t\right),$$

where the symmetric deformation rate tensor $D\left(t\right)=\frac{1}{2}\left[L+L^{T}\right]$ and $\eta_{s}$ is the solvent viscosity. The model parameters $\Theta =[\eta_{p},\eta_{s},\tau_{D},\tau_{R},\chi_{max},\beta ]$ define a specific Rolie-Poly fluid. Further details about the Rolie-Poly model can be found elsewhere, e.g., [28,29].

## 3. Methodology

**Figure 1:**
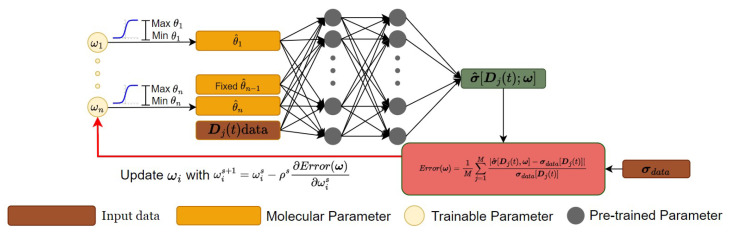
A flowchart of a DNN-based numerical solver for inversely computing molecular parameters.

A general deep learning method for computing molecular parameters of viscoelastic constitutive models by solving inverse problems is proposed and outlined in Figure 1. This method comprises two components: (1) a Deep Neural Network (DNN) serving as an alternative representation for modeling the molecular constitutive relationship; (2) a DNN-based numerical solver for inversely determining the molecular parameters Θ of the constitutive model σ(t)=Ψ[D(t);Θ] from a set of known stress tensor σ and strain rate tensor D data through gradient descent, as illustrated below.

### 3.1. DNN Representation of Molecular Constitutive Model

A fully-connected neural network is first trained by exploring the parameter space of a given molecular constitutive model, e.g., the Rolie-Poly model presented in this work. It comprises an input layer, a number of N−1 hidden layers and an output layer. The variables in the input layer X include a series of specified molecular parameters $\Theta =[\theta_{1},\theta_{2},\ldots \theta_{n}]$ and strain rate tensor D. The transformation of the variables from the (k−1)th to the *k*th layer can be expressed as follows:

(7) $$L_{k}\left({\hat{X}}^{k-1}\right):={\hat{y}}^{k}=\alpha^{k}\left(W^{k}{\hat{X}}^{k-1}+b^{k}\right),$$

where k=1,…,N and ${\hat{X}}^{0}=X$; $W^{k}\in R^{N_{k}\times N_{k-1}}$ are the weights of the connections between the *k*th and the (k−1)th layer, $b^{k}\in R^{N_{k}}$ is the biases term of the *k*th layer. $\alpha^{k}$ is a nonlinear activation function, e.g., *Tanh*, applied to each component of the transformed vector from each layer to make the transformation nonlinear. Note that no nonlinear activation function is used in the transformation of the last layer $L_{N}$. After the *N*th transformation, the output of the DNN can be expressed as the composition of $L_{k}$ functions

(8) $$\sigma_{\Xi }\left(X\right)=L_{N}\circ \ldots \circ L_{k}\circ \ldots \circ L_{1}\left(X\right),$$

where $\Xi ={\left\{W^{k},b^{k}\right\}}_{k=1}^{N}$ represents the trainable parameters of the DNN. It will be trained by minimizing the loss function defined, as follows:

(9) $$Loss=\frac{1}{M}\sum_{j=1}^{M}{\left(\sigma \left[D_{j}\left(t\right);\Theta \right]-\hat{\sigma }\left[D_{j}\left(t\right);\Theta \right]\right)}^{2}+\xi \sum_{w_{i}\in W}w_{i}^{2},$$

where the first term is a measure of the total errors between the DNN output, $\hat{\sigma }\left[D\left(t\right);\Theta \right]$, and the theoretical value, σ[D(t);Θ], predicted by the target constitutive equation; *M* is the total number of strain rate data points sampled in the training. The second term is L2 regularization and ξ is L2 regularization rate for weight functions to prevent over fitting. The minimization could be formulated as an optimization problem and solved by a stochastic gradient descent (SGD) algorithm, within which the trainable parameters are updated iteratively as the following:

(10) $$\Xi^{m+1}=\Xi^{m}-\eta_{l}\nabla_{\Xi^{m}}J^{m}\left(\Xi \right),$$

where $\eta_{l}>0$ is the learning rate and *m* represents the *m*th iteration. A variant of the SGD optimization algorithm, ADAM [30], will be used here.

In general, such a DNN could approximate complex functions to any desired accuracy as long as a sufficient number of hidden units and data are available [31,32]. The differentiability of the deep neural network is also advantageous for solving inverse problems. The DNN will be trained with sufficient accuracy to accurately represent the target constitutive model across its molecular parameter space and the range of strain rates encountered during training.

### 3.2. A DNN-Based Numerical Solver for Inversely Computing Molecular Parameters

Figure 1 shows a flowchart of a DNN-based numerical solver for inversely computing molecular parameters. For any given set of the viscoelastic stress $\sigma_{data}\left[D_{j}\left(t\right)\right]$ obtained under a known deformation rate $D_{j}\left(t\right)$ where j∈[1,M], initial trial parameters $\omega =[\omega_{1},\omega_{2}\ldots ,\omega_{i}\ldots ,\omega_{n}]$ are randomly selected over their parameter space. The trial parameters ω are then converted to the model parameter Θ with its *i*th parameter defined as

(11) $$\;\hat{\theta_{i}}=\left(Max\;\theta_{i}-Min\;\theta_{i}\right)g\left(\omega_{i}\right)+Min\;\theta_{i}$$

where $Min\;\theta_{i}$ and $Max\;\theta_{i}$ are the minimum and maximum value of the *i*th molecular parameter, respectively, between which the DNN has been pre-trained with a sufficient accuracy to represent the molecular constitutive model; the function *g* is defined as

(12) $$g\left(\omega_{i}\right)=\frac{1}{1+e^{-\omega_{i}}}$$

and is a Sigmoid function with the monotonic property over its variable range of $g\left(\omega_{i}\right)\in \left[0,1\right]$. The stress tensor $\hat{\sigma }\left[D_{j}\left(t\right),\omega \right]$, as a function of the known deformation rate $D_{j}\left(t\right)$ and the trial parameters ω, is then computed by the pre-trained DNN. The outcomes are measured by an error function, defined as

(13) $$Error\left(\omega \right)=\frac{1}{M}\sum_{j=1}^{M}\frac{|\hat{\sigma }\left[D_{j}\left(t\right),\omega \right]-\sigma_{data}\left[D_{j}\left(t\right)\right]|}{\sigma_{data}\left[D_{j}\left(t\right)\right]}.$$

The inverse problem is solved by minimizing the error functions using the gradient descent method, within which the gradient is computed through automatic differentiation [33,34]. In the *s*th optimization iteration, the *i*th parameter $\omega_{i}$ is updated as

(14) $$\omega_{i}^{s+1}=\omega_{i}^{s}-\rho^{s}\frac{\partial Error(\omega )}{\partial \omega_{i}^{s}}$$

where $\rho^{s}$ is an iteration parameter and would decrease by an adjustable factor when the minimization of the error function is nearly saturated in order to refine the convergence. The above optimization iteration is repeated until a convergence criterion is satisfied as

(15) $$|\omega^{s+1}-\omega^{s}|<\epsilon$$

where ϵ is set in advance as a very small value. Moreover, in order to validate the independence of the solution from the initial conditions, the inverse problem could be solved by randomly selecting several different sets of the initial trial parameters ω.

## 4. Results and Discussion

### 4.1. Training DNNs for Modeling Entangled Polymer Solutions

To illustrate the training of DNNs for modeling entangled polymer aqueous solutions, Table 1 presents the parameter variations of the Rolie-Poly model. The solvent viscosity $\eta_{s}$ is typically treated as a constant and, in this illustrative case, set to the viscosity of water [35,36]. Training data were generated by sampling the model’s parameter space. For the input layer, each molecular parameter was uniformly sampled either in a linear or logarithmic scale across its range of variation. At least ninety data points were uniformly distributed in logarithmic scale under simple shear flow across a wide shear rate range from $10^{-4}$ to $10^{4}$. For each set of the sampled model parameters and shear rate, the corresponding output layer data were obtained by calculating steady viscoelastic stresses from the Rolie-Poly model. The dataset consists of 15,000 sets of sampled data, with 10,000 for training, 3000 for validation, and 2000 for testing. The DNN’s learning rate was adjusted around 0.001 to achieve satisfactory learning convergence. The training was conducted at least three times using a DNN with varying sizes in terms of hidden layers and neurons, as presented in Table 2. The results indicate the lowest relative error between viscoelastic stresses computed by the DNNs and the original model. The DNN with the lowest overall relative error features 5 hidden layers and 192 neurons, and will be utilized in subsequent studies.

### 4.2. Validation of Convergence

**Figure 2:**
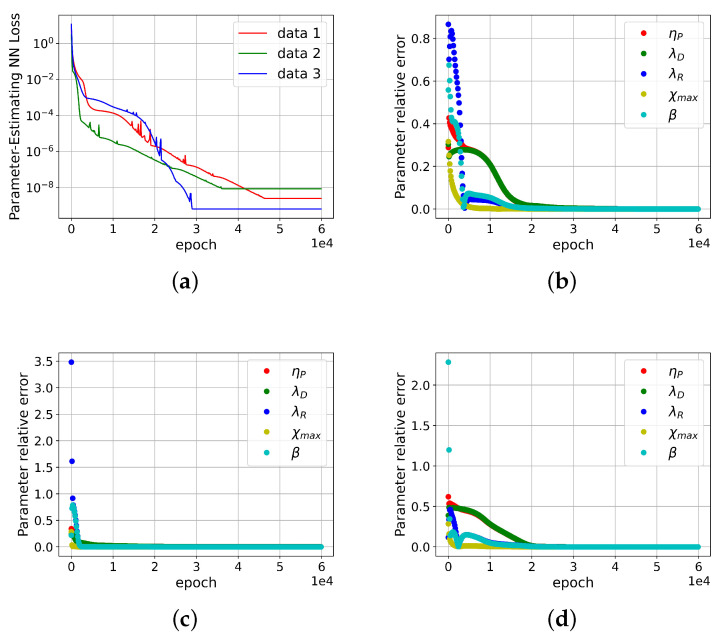
The convergence of the DNN-based solver for inversely computing molecular parameters. The corresponding viscoelastic stresses over a range of shear rates are calculated from the trained DNN. (**a**) the loss of the DNN-based solver is plotted against a number of iterations; (**b**–**d**) the relative errors of the estimated molecular parameters are plotted against a number of iterations for the testing samples 1, 2 and 3, respectively.

To validate the DNN-based solver for inversely computing molecular parameters, three sets of model parameters were arbitrarily sampled across the range specified in Table 1, and these sets were treated as the target solutions to be obtained. The corresponding viscoelastic stresses over a range of shear rates were calculated using the trained DNN and employed as inputs for solving the inverse problem. Initial values for the model parameters were randomly generated within the range defined in Table 1. As depicted in Figure 2, the loss of the DNN-based solver, along with the relative error in the estimated molecular parameters, rapidly converges with an increasing number of iterations. This ensures accurate solution of the inverse problem. However, the convergence rate for the model parameters $\eta_{P}$ and $\lambda_{D}$ is relatively slower compared to other parameters, owing to their significantly wider range of variation.

**Figure 3:**
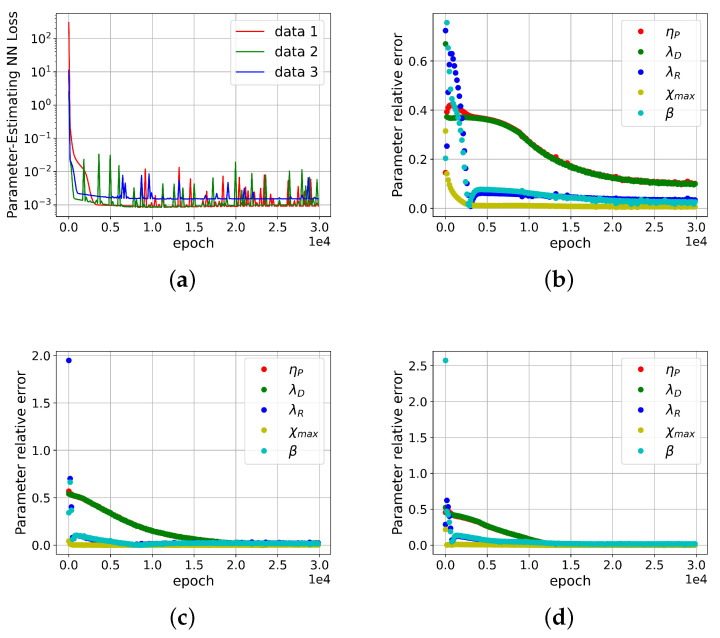
The convergence of the DNN-based solver for inversely computing molecular parameters. The corresponding viscoelastic stresses over a range of shear rates are calculated from the Rolie-Poly model. (**a**) is the Parameter-Estimating loss throughout the training process with randomly sampled data. (**b**–**d**) represent the molecular parameter relative error curves for data index 1, 2 and 3, respectively.

Instead of utilizing the trained DNN, the corresponding viscoelastic stresses over a shear rate range could be directly computed using the Rolie-Poly model, and these computed stresses can then be employed as inputs for solving the inverse problem. Following the same procedure as described above for inversely determining the molecular parameters, the loss of the DNN-based solver and the relative error of the estimated molecular parameters are plotted against the number of iterations in Figure 3. In comparison with Figure 2, reasonable convergence is achieved. As depicted in Figure 3b, the relative error for the model parameters $\eta_{P}$ and $\lambda_{D}$ is approximately 10%. This error could potentially be further minimized by enhancing the accuracy of the DNN’s approximation of the Rolie-Poly model.

### 4.3. Effects of Input Data Noise

**Figure 4:**
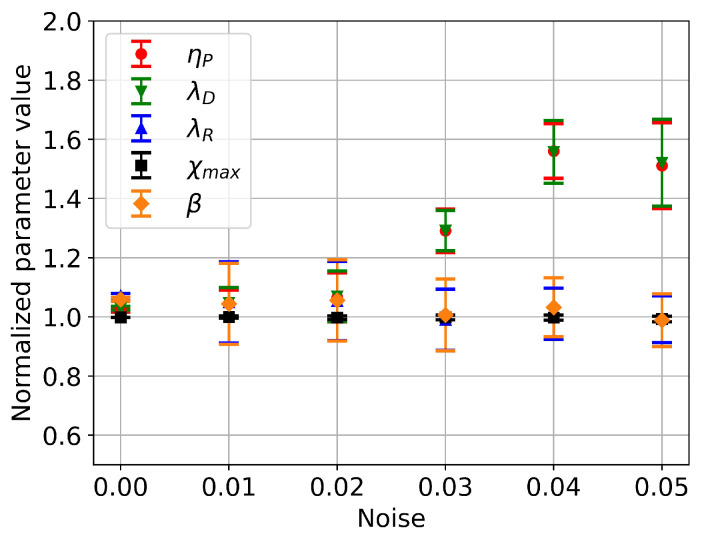
A plot of the normalised mean and standard deviation of the model parameters against the noise level.

To assess the impact of stress data noise on molecular parameter estimation, white noise was added to the viscoelastic stress data, causing the input data to randomly deviate from their original values by a certain maximum percentage. For a given noise level, eight sets of such noisy input data were generated. Each set of noisy input data underwent ten iterations of the DNN-based solver, employing randomly generated initial values across the parameter range, to validate solution consistency. The mean and standard deviation of each model parameter were subsequently calculated and presented in Figure 4. In this representation, the estimated parameters are normalized by their solutions computed using noise-free input data. Error bars correspond to the standard deviation of the means. Across the noise levels ranging from 0 to 0.02, mean value changes align with their respective standard deviations and lack statistical significance. Nevertheless, as the noise level escalates to 0.03, mean solution values for parameters $\eta_{P}$ and $\lambda_{D}$ deviate statistically from the original ones. With further noise escalation beyond 0.03, the mean solution values of parameters $\eta_{P}$ and $\lambda_{D}$ diverge further from the original solutions and become saturated from a noise level of 0.04 onwards. The standard deviation also experiences a minor increase. Throughout the noise level variation range, mean solution values for parameters $\lambda_{R}$, $\chi_{max}$, and β remain statistically indistinguishable from their original solutions. These outcomes mirror the information presented in Table 1, indicating that the variation ranges of parameters $\eta_{P}$ and $\lambda_{D}$ are approximately two orders of magnitude greater than those of parameters $\lambda_{R}$, $\chi_{max}$, and β, rendering them substantially more sensitive to stress data noise.

### 4.4. Validation by Completely Monodisperse Entangled Polymer Solution

**Figure 5:**
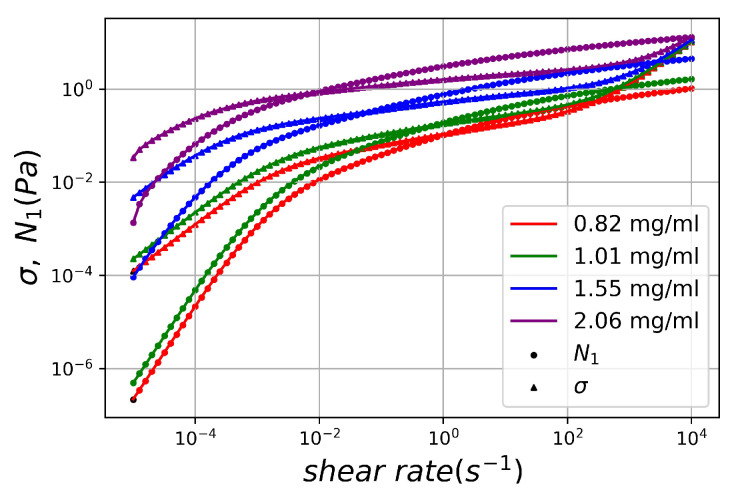
Comparison of the original input steady stress (σ, $N_{1}$ in symbols) vs. shear rate data with the data (in lines) calculated by the Rolie-Poly model with the parameters inversely solved by the DNN-based solver for the completely monodisperse ultrahigh-molecular-weight linear lambda DNA solutions.

Banik et al. [35] recently presented molecular parameters deduced from the linear viscoelastic data of entirely monodisperse ultrahigh-molecular-weight linear lambda DNA solutions across various entangled concentrations. To validate the DNN-based solver for inverse calculation of molecular parameters, steady shear stress (σ) and the first normal stress difference ($N_{1}$) were generated through the Rolie-Poly model, utilizing the aforementioned extracted molecular parameters, spanning a shear rate range of $10^{-4}$ to $10^{4}$. By utilizing the steady stress of the DNA solution as input data, the DNN-based solver was employed for the inverse problem solution, employing the gradient descent method as an optimizer. For each DNA sample concentration, model parameters were resolved ten times by the DNN-based solver using different initial trial values of the Rolie-Poly model parameter, randomly chosen within the parameter range. Averaged solutions for each model parameter, stemming from ten distinct sets of initial trial values, along with their respective standard deviations, were tabulated in Table 3. The table also encompasses mean relative errors of the steady first normal stress difference ($N_{1}$) and the steady shear stress (σ) over approximately 91 shear rates. Results indicate minimal impact of initial trial parameter values on the averaged solutions, with small standard deviations compared to the averaged values derived by the DNN solver. Notably, the DNN-based solver offers solutions that closely align with extracted molecular parameters. As depicted in Figure 5, the curves for steady viscoelastic stress (σ, $N_{1}$) versus shear rate, computed using model parameters obtained through the inverse problem solution, agree remarkably well with those predicted by the Rolie-Poly model using the originally extracted parameters. Examination of concentration scaling for $\eta_{P}$ and $\lambda_{D}$ reveals power law exponents of 6.63 and 4.38, respectively. These outcomes harmonize with exponents of 6.7±0.3 and 4.7±0.3 estimated from linear viscoelastic data of entirely monodisperse lambda DNA solutions using the time-concentration superposition method [35,36]. An intriguing observation is that the DNN-based solver not only accurately derives model parameter solutions, but also effectively deciphers the power-law concentration scaling of DNA solution’s material function. It is important to note that this concentration scaling was not explicitly programmed into the DNN or the Rolie-Poly model; instead, it was implicitly learned by the DNN through sampling across the Rolie-Poly model’s material parameter space.

## 5. Conclusions

A general deep learning method for computing molecular parameters of a viscoelastic constitutive model is proposed. It utilizes a DNN as an alternative representation for modeling the molecular constitutive relationship and a DNN-based numerical solver for inversely computing the molecular parameters of the pre-trained constitutive model. Without losing much generality, the method was validated by considering the Rolie-Poly model for modeling the linear and non-linear steady rheometric properties of entangled polymer solutions in a wide range of concentrations. The results show that as long as the proposed DNN could represent the Rolie-Poly model with sufficiently high accuracy, the DNN-based numerical solver would rapidly converge to its solution while inversely computing molecular parameters. The solution of the DNN-based solver is robust against small white noise disturbances to the input stress data. However, if the input stress significantly deviates from the original stress, the DNN-based solver could readily distinguish such a difference and converge to a different solution. This demonstrates the effectiveness of the numerical solver for inversely computing molecular parameters. The molecular parameters determined through the DNN-based numerical solver, utilizing the steady stress data of fully monodisperse linear lambda DNA solution, not only precisely replicate the steady stress behavior across an extensive shear rate spectrum and multiple concentrations, but also effectively capture the power-law concentration scaling phenomenon with almost identical scaling exponents as those inferred from experimental findings.

In principle, the proposed DNN-based solver for inverse calculation of material functions in constitutive models holds the potential to be extended to other complex fluids. The physics-informed DNN can be further trained using experimental and computational simulation data to incorporate additional factors expressed through their material functions. These factors could encompass temperature, pressure, molecular mass and distribution, composition, solvent quality, and more. This expanded approach allows the DNN to encapsulate more intricate multiple-scale dynamic mechanisms, enabling quantitative predictions. Therefore, in conjunction with conventional methods, a synergistic model and data-driven strategy is poised to emerge. This approach will facilitate the optimization of molecular and formulation designs for diverse complex fluids, achieved by solving inverse problems across a wide spectrum of industrial products and intelligent manufacturing processes.

## Figures and Tables

**Table 1:** A range of parameter variation of the Rolie-Poly model.

Parameter	Variation Range (SI Unit)
$\eta_{P}$	1∼6000 Pa·s
$\lambda_{D}$	1∼5000 s
$\lambda_{R}$	1∼50 s
$\chi_{max}$	15∼20
β	1∼25

**Table 2:** The lowest relative error of the DNNs with different sizes.

	Neurons	64	128	192	256
Hidden Layers					
2		2.31%	1.65%	1.46%	1.49%
3		2.34%	1.45%	1.41%	1.43%
4		2.39%	1.97%	1.74%	1.38%
5		2.61%	1.56%	**1.33%**	1.54%
6		2.26%	1.49%	1.57%	1.65%

**Table 3:** The molecular parameters inversely solved by the DNN-based solver for the lambda DNA solution over various entangled concentrations [35].

	Concentration	0.82 mg/mL		1.01 mg/mL		1.55 mg/mL		2.06 mg/mL	
Parameter		Extracted	DNN Solution	Extracted	DNN Solution	Extracted	DNN Solution	Extracted	DNN Solution
$\eta_{P}$		12.4	12.53 ± 0.02	22.6	22.58 ± 0.02	474.4	474 ± 1	4811.7	4784.2 ± 0.4
$\lambda_{D}$		87.0	88.00 ± 0.09	109.0	109.00 ± 0.01	1006.0	998 ± 4	4092.0	4095.10 ± 0.02
$\lambda_{R}$		11.0	10.7 ± 0.1	14.0	13.00 ± 0.08	21.0	20.5 ± 0.2	29.0	31.70 ± 0.02
$\chi_{max}$		18.0	19 ± 2	18.0	17.2 ± 0.7	18.0	19 ± 1	18.0	17.500 ± 0.004
β		20.0	19.5 ± 0.3	13.0	12.20 ± 0.06	5.00	5.00 ± 0.05	1.0	1.000 ± 0.002
$Error\;of\;N_{1}$			0.0076		0.0025		0.0017		0.005
Errorofshearstress			0.0037		0.0017		0.0019		0.0035

## Data Availability

The data presented in this study are available on request from the corresponding authors.
